# Diosgenin, a Novel Aldose Reductase Inhibitor, Attenuates the Galactosemic Cataract in Rats

**DOI:** 10.1155/2017/7309816

**Published:** 2017-09-05

**Authors:** Lixia Ji, Lixia Cheng, Zhihong Yang

**Affiliations:** ^1^Department of Pharmacology, School of Pharmacy, Qingdao University, Qingdao, China; ^2^Department of Endocrinology, Weifang People's Hospital, Weifang, China

## Abstract

**Objective:**

To seek efficient aldose reductase inhibitors (ARIs) with excellent in vitro and in vivo biological activities against rat galactosemic cataract.

**Methods:**

The method was firstly optimized to screen strong ARIs from nonoriented synthetic compounds and natural extracts. Then, diosgenin was assessed on osmotic expansion of primarily cultured lens epithelial cells (LECs) induced by galactose (50 mM). Diosgenin was administered to galactosemic rats by oral (100 and 200 mg/kg) or direct drinking (0.1%) to evaluate its anticataract effects.

**Results:**

Diosgenin was found as the strongest ARI with IC_50_ of 4.59 × 10^−6^ mol/L. Diosgenin (10 *μ*M) evidently inhibited the formation of tiny vacuoles and upregulation of AR mRNA in LECs. In vivo, diosgenin delayed lens opacification, inhibited the increase of ratio of lens weight to body weight, and decreased AR activity, galactitol level, and AR mRNA expression, especially in the diosgenin drinking (0.1%) group.

**Conclusions:**

Diosgenin was an efficient ARI, which not only significantly decreased the LECs' osmotic expansion in vitro but also markedly delayed progression of rat galactosemic cataract in vivo. Thus, diosgenin rich food can be recommended to diabetic subjects as dietary management to postpone the occurrence of sugar cataract, and diosgenin deserves further investigation for chronic diabetic complications.

## 1. Introduction

Diabetes, a global epidemic noncommunicable disease, is predicted to rise to 552 million by 2030 [1]. Although we have obtained great advances in hypoglycemic agents in the past several decades, the long-time perfect glycemic control seems not to be always possible. Over ten years later, diverse chronic complications including cataract gradually arise [2]. Cataract, characterized by painless and progressive opacification of lens, is still the leading cause of visual impairment, and now the only efficient treatment is surgery. Diabetic cataract differs from the age-related cataract; the robust factor is not the oxidative stress but the osmotic expansion of lens. Osmotic expansion is a relatively reversible procession occurring in early stage, which triggers the rapid onset and accelerates the severity of true cataract [3, 4].

The lens, an almost perfect organ, lives in hypoxic microenvironment. The front is aqueous humor and the rear is vitreous liquid, which has neither vascular nutrition nor nerve innervations. In physiological condition, over 80% glucose enters the glycolysis pathway and almost no glucose enters the polyol pathway, the key cascade of sugar cataract [3, 5]. However, in hyperglycemia, hexokinase would be almost saturated especially as blood glucose level is over 11.1 mmol/L; soon after AR has been fully activated, more than 30% glucose enters polyol pathway and is converted to sorbitol. Furthermore, sorbitol is poorly metabolized by sorbitol dehydrogenase, and it does not readily diffuse across the cell membranes due to poor liposolubility; so a large amount of hypertonic sorbitol accumulates within lenticular cells and leads to the gradual swelling and even membrane rupture [3]. The hydration of lenticular cells was firstly discovered in the lens equatorial zone and cortical fiber cells, then in LECs below the anterior capsule [6]. In fact, AR is primarily present in LECs and then gradually transfers to fiber cells at the equatorial area.

In past decades, the once most promising ARIs such as sorbinil, tolrestat, zopolrestat, and ponalrestat were withdrawn from clinical trials because of unsatisfactory efficacy and severe side effects [4, 7]. Now, the only commercial AR inhibitor, epalrestat, has been commercially available in Japan to treat nephropathy complication for several years and has been recently permitted for marketing in China and India [2, 8]. Another promising AR inhibitor is Kinostat, which shows strong effects against cataract on diabetic dogs, and may acquire FDA approval in 2017 [9]. Recently, the plant-derived compounds have also obtained considerable attention; some flavones even show remarkable AR inhibitory activity with IC_50_ at 10^−6^ mol/L level [10–12]. Here, we report a new-screened ARI, diosgenin, and its effects against sugar cataract in vitro and in vivo.

## 2. Materials and Methods

### 2.1. Preparation of AR from Normal Rat Lenses

The whole lenses were instantly removed from normal Sprague Dawley (SD) rats by posterior capsule method after being sacrificed. Each lens was homogenized in 5 × volumes of 50 mM ice-cold phosphate buffer saline (PBS, pH 7.2, with 5 mM 2-mercaptoethanol), then centrifuged at 15,000*g* at 4°C for 30 min. The supernatant was collected and used as an AR source. Lens supernatant should be prepared freshly due to the quick inactivation of the AR enzyme.

### 2.2. Optimization of ARIs Screening Method and Screening of Efficient ARIs

In the first step of polyol pathway, glucose reduction is accompanied by the conversation of NADPH to NADP. While, only NADPH has an obvious spectral absorption around 340 nm and NADP has none; thus, the decrease of OD_340nm_ can represent the consumption of NADPH. Therefore, we detect the change of OD_340nm_ before and after reaction to screen the effective ARIs or evaluate AR activity. The incubation system contains lens supernatant as an AR enzyme, 0.16 mmo1/L DL-glyceraldehyde as substrate, 1.0 mmo1/L NADPH·Na_4_ as coenzyme, and 0.1 mol/L PBS (pH = 6.2). The incubation mixture was minimized to a total volume of 200 *μ*L and ongoing in a 96-well ultraviolet plate. In order to ensure accuracy of the screening method, we made all the combinations of the above components and scanned them from 200 nm to 999 nm to validate the OD_340nm_ absorption peak of NADPH.

As to the ending method, AR, a 36 kD protein enzyme, is easily inactivated by the change of pH value and temperature. Acidation, alkalization, and heating can easily lead to the denaturation of AR, so we think that perhaps cooling is a desirable ending method. After strict comparisons among different cooling methods, we found that the best convenient way was incubation at −20°C for 5 minutes. The appropriate reactive degree is about 80%; the above freezing ending method reserves a chance for the reaction to restart once it is less than 60%. Epalrestat was chosen as the positive ARI; we collected 224 candidates including 180 nonoriented synthetic compounds and 44 natural extracts, all these samples were primarily dissolved in DMSO at 10^−3^ mol/L and then were diluted to a final concentration of 10^−6^ mol/L for ARIs screening.

### 2.3. Primary Culture of LECs of Beagle dogs

Each whole eyeball was immediately removed and soaked into 75% ethanol after sacrificing the beagle dog; then we carefully isolated the intact lens and dissected the capsule into two halves from the equatorial region. All the anterior parts were collected and washed twice by using the artificial aqueous humor. Then, we added 1 ml fetal bovine serum (FBS) to infiltrate them, cut them into tiny pieces and moved them into a flask, and gently shook them to scattered evenly on the flask bottom. In order to enhance the adhesion, the flask was incubated in an erect position for 4 hours at 37°C in presence of 5% CO_2_, followed by positioning it horizontally. After 24 hours, we observed whether new LECs were growing below or around the capsule fragments and added 5 ml of Dulbecco's Modified Eagle's Medium (DMEM, containing 1.0 mM glucose) supplemented with 15% FBS. Each step should be done gently and we tried to avoid shaking during primary cultivation. When the new growing multilayer LECs accumulated around the tissue fragment, they should be digested by 0.1% trypsin for passage. The LECs from the 3rd to 8th passage were used for further studies.

### 2.4. Induction of LECs Osmotic Expansion and Effects of Diosgenin

The groups were designed as follows: normal group, control group (galactose 50 mM), Epal group (epalrestat 5 *μ*M), and two DG groups (5 and 10 *μ*M). LECs (the 3rd–8th passage) were firstly harvested and seeded on 6-well culture plates (2 × 10^4^ per well). 24 hours later, galactose was added to control, Epal and DG groups, epalrestat and diosgenin were, respectively, added to Epal and DG groups. After 48 hour incubation, we observed the LECs morphology under an inverted microscope and harvested them, promptly extracted the total RNA by TRIzol reagent and measured the AR mRNA level by real time RT-PCR.

### 2.5. Induction of Rat Galactosemic Cataract and Administration of Diosgenin [13]

In brief, 21 day normal male SD rats were randomly divided into 5 following groups (*n* = 10): normal group, control group, and three DG groups (diosgenin-100, 200, and 0.1%). All the rats ate normal chew; rats of the normal group drank the purified water, but rats of other groups had ad libitum access to 12.5% galactose solution in the first 7 days, then to 10% galactose solution up to the end of 15 days. Rats of the DG (100, 200) groups were orally treated with two dosages of 100 and 200 mg/kg, respectively; rats of the DG (0.1%) group directly drank 0.1% diosgenin solution mixed with galactose. All animal procedures were performed in accordance with the ARVO Statement for the Use of Animals in Ophthalmic and Vision Research and approved by the Institutional Animal Ethics Committee.

### 2.6. Measurement of Lens Opacity

Lens opacity was monitored by a handed slit lamp (Kowa SL-15, Japan) at the day of 3, 6, 9, 12, and 15, respectively. Each time, the pupils were fully dilated with a topical ophthalmic solution containing tropicamide 5% and phenylephrine hydrochloride 5%. The anterior segment including the lens was observed and photographed in both eyes of all the rats. Lens opacification was scored as follows [14]: grade 0, clear normal lenses; grade 1, vacuoles, located in the cortex, cover less than 1/3 of the lens anterior segment, and forming a subcapsular cataract; grade 2, vacuoles cover approximately 2/3 of the lens anterior segment; grade 3, diffuse opacity in cortex with/without some vacuoles; grade 4, diffuse opacity in cortex and moderate nuclear opacity; and grade 5, mature milky cataract is observed as a dense opacity in both cortex and nucleus. At the end, all the lenses were carefully isolated, weighted, and promptly dipped in liquid nitrogen to preserve them for one month until further use.

### 2.7. AR Activity, Galactitol Level, and AR mRNA Expression in Lenses

Each lens was homogenized according to the above AR preparation method; the supernatant was taken to detect the AR activity. One unit of AR enzyme activity is defined as the amount of enzyme catalyzing the oxidation of 1 *μ*mol NADPH per hour per 100 mg protein [13]. The total RNA of lens was extracted by the TRIzol reagent; AR mRNA level was detected by real time RT-PCR method.

Lenticular galactitol level was detected as follows [13]: 80 *μ*L of lens homogenate was fully mixed with 20 *μ*L of 20% TCA, centrifuged at 5,000*g* for 10 minutes at 4°C, and then the supernatant was collected to determine the galactitol level. The reaction system consisted of the lens supernatant, 12.5 mM periodic acid, 12.5 mM sodium arsenite, and 0.2% chromotropic acid. The absorption peak was recorded at 570 nm to calculate the level of galactitol

### 2.8. Statistical Analysis

All data were expressed as mean ± SD, cataract grade was analyzed by Mann–Whitney test, and other data were evaluated with one-way ANOVA (two-tailed test). The *P* value less than 0.05 was considered to be statistically significant.

## 3. Results

### 3.1. Optimization of ARIs Screening Method

**Figure 1:**
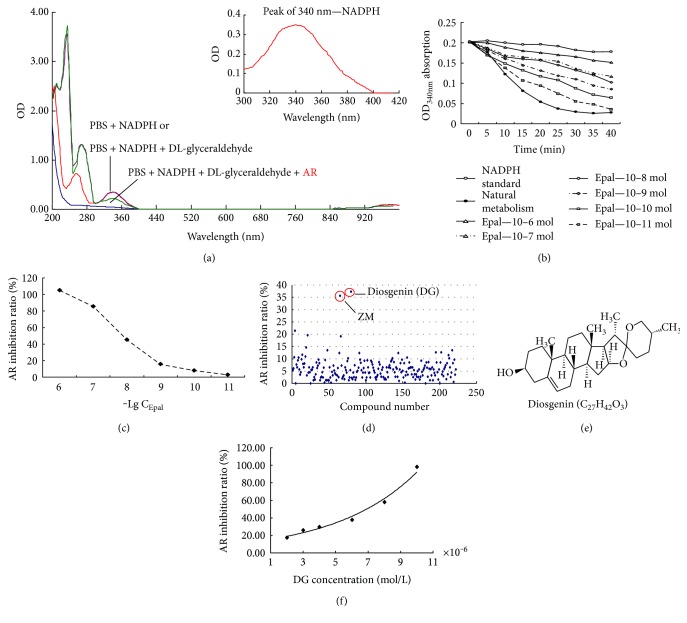
Optimization of ARIs screening method and screening results of ARIs. (a) The scanning plots of different combinations of all components from 200 nm to 999 nm. (b) Inhibition of epalrestat (10^−11^–10^−6^ mol/L) on normal rat lens AR with good dose-dependent manner. (c) Dose-response curve of epalrestat on rat lens AR based on the result of 10 minutes, IC_50_ = 1.32 × 10^−8^ mol/L. (d) ARIs screening results of 224 candidates, diosgenin was the strongest ARI. (e) Chemical structure of diosgenin, MW = 414.61. (f) Dose-response curve of diosgenin on rat lens AR, IC_50_ = 4.59 × 10^−6^ mol/L.

As shown in Figure 1(a), PBS and lens supernatant had no spectral absorption around 340 nm, and the absorption peak of NADPH was exactly at 340 nm. The fully overlapped absorption curve in the merged figure indicated DL-glyceraldehyde did not influence OD_340nm_ absorption of NADPH. Only after addition of AR for several minutes did a remarkable decrease of OD_340nm_ absorption appear. These results clearly demonstrated that NADPH had an absorption peak at OD_340nm_, and stimulated AR of normal rat lens could consume NADPH and leads to the decrease of OD_340nm_ absorption.

### 3.2. Verification of ARIs Screening Method and Efficient ARIs Screening

Gradient epalrestat (10^−11^–10^−6^ mol/L) had significant inhibition on AR activity of normal lens (Figure 1(b)). The dose-response curve of epalrestat was made based on the inhibitory rate of 10 minutes; the IC_50_ of epalrestat was 1.32 × 10^−8^ mol/L (Figure 1(c)), which was comparable to other studies [15, 16]. Epalrestat was known as the positive ARI; this result fully verified the accuracy of the new optimized ARIs screening method. Among 224 candidates, most had no inhibitory effects on lens AR besides diosgenin and ZM (a Chinese traditional recipe), and diosgenin is the stronger ARIs (Figure 1(d)). Diosgenin is a widespread natural extract with definite chemical structure (Figure 1(e)), its strong inhibition on rat lens AR was in a good dose-dependent manner, and the IC_50_ value was 4.59 × 10^−6^ mol/L in the present study (Figure 1(f)).

### 3.3. Morphological Feature of Primarily Cultured LECs

**Figure 2:**
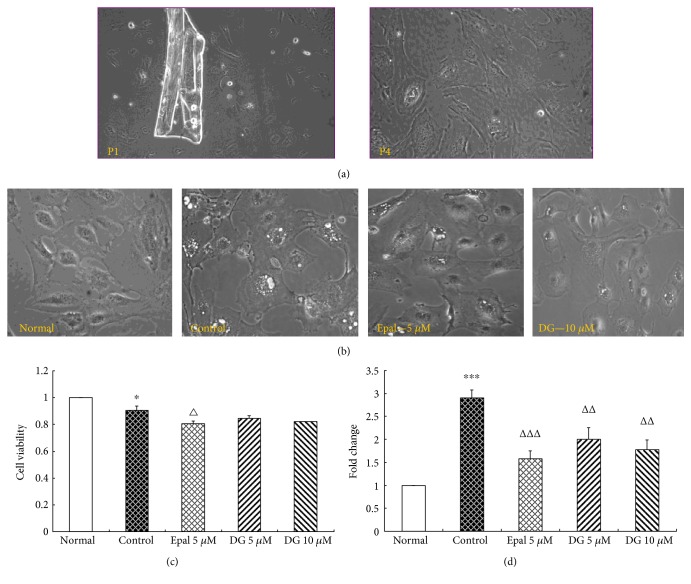
Effects of diosgenin on LECs osmotic expansion induced by 50 mM galactose. (a) Primary culture of beagle dog LECs, P1 and P4 represent the 1st and 4th passage, respectively. (b) Effects of diosgenin (DG) on LECs' osmotic expansion (tiny vacuoles) induced by galactose (50 mM). (c) Effects of DG on LECs' cellular viability. (d) Effects of DG on AR mRNA expression. Values are mean ± SD, ^∗^*P* < 0.05, ^∗∗∗^*P* < 0.001 versus the normal group, ^△^*P* < 0.05, ^△△^*P* < 0.01, and ^△△△^*P* < 0.001 versus the control group.

As shown in Figure 2(a), some tiny lens capsule tissues were tightly adhered to the bottom of the flasks, 48 hours later, and some growing LECs were clearly seen below the semitransparent lens capsule pieces. Numerous LECs were growing around the capsule tissue in good status; most of them were oval or irregular polygonal with large visible nucleolus, abundant cytoplasm, and little tentacles. The tightly adhered LECs had rapid proliferative ability during the following passage; several elongated fiber cells could exist after the 10th passage.

### 3.4. Morphologic Characteristics, Cell Viability, and AR mRNA Expression of LECs

As shown in Figures 2(b), 2(c), and 2(d), 50 mM galactose induced the obvious osmotic expansion in LECs, plenty of tiny vacuoles existed in the cytoplasm especially around nucleus, LECs viability decreased significantly, and AR mRNA expression was upregulated (2.85-fold). While after 48 hour coincubation with epalrestat or diosgenin, the numbers and value of vacuoles obviously reduced; the upregulation of AR mRNA was also significantly restrained by epalrestat and diosgenin; and LECs viability decreased only in the epalrestat (5 *μ*M) group.

### 3.5. Formation of Sugar Cataract and the Ratio of Lens Weight to Body Weight

**Figure 3:**
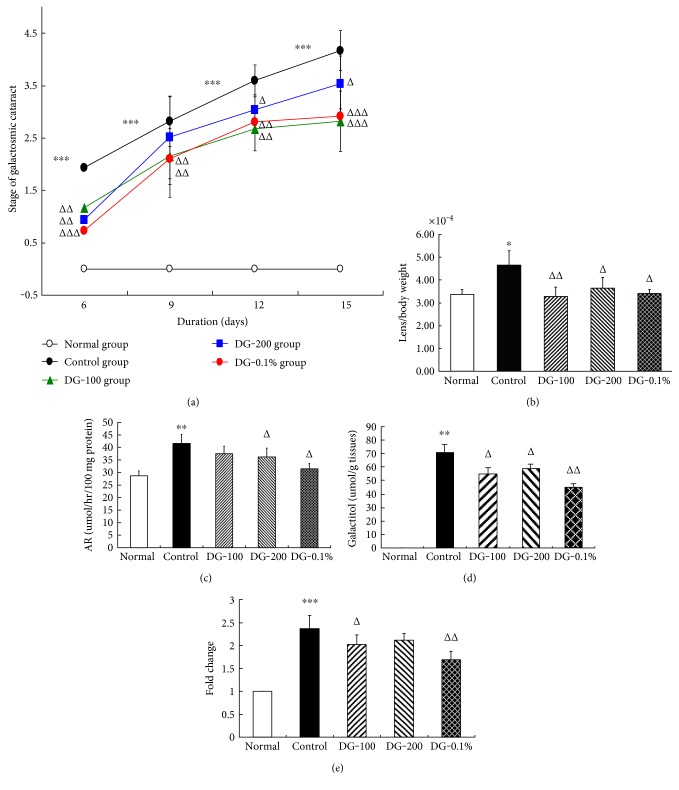
Effects of diosgenin on rat galactosemic cataract in vivo. (a) Effects of diosgenin on lens opacification evaluated using a slit lamp. (b) Effects of diosgenin on the ratio of lens weight to body weight, which represents the severity of lens osmotic expansion. (c) Effects of diosgenin on lens AR activity of galactosemic rats. (d) Effects of diosgenin on lens galactitol level of galactosemic rats. (e) Effects of diosgenin on lens AR mRNA expression of galactosemic rats. Values are mean ± SD, ^∗^*P* < 0.05, ^∗∗^*P* < 0.01, ^∗∗∗^*P* < 0.001 vs the normal group, ^△^*P* < 0.05, ^△△^*P* < 0.01, ^△△△^*P* < 0.001 versus the control group. DG-100 and DG-200 mean diosgenin were given orally (100 and 200 mg/kg); DG-0.1% represents the rats who directly drank 0.1% diosgenin solution.

As shown in Figure 3(a), in the whole duration, all the lenses of the normal group were clear and at grade 0, lens opacification of the control group increased by almost one grade every 3 days, and cataract grades of other three DG groups were always lower than the control level. On the 3rd day, so many tiny vacuoles appeared below the anterior capsule in the cataract model, and almost no vacuoles existed in the lenses of three DG groups. By the 6th day, the average cataract grade of the control group was 1.87; however, that of the DG-100 and 200 groups were only 1.07 and 0.84, respectively, and the lowest level was 0.67 in the DG-0.1% group. Then lens opacification of the control and DG treating groups gradually increased in the following days. At the end, more than 80% lenses of the control group were milky (grade 4), the average levels of the DG-100, 200, and DG-0.1% groups were 2.63, 3.45, and 2.72, respectively, and the lowest level (2.63) was in the DG-100 group. On the whole, diosgenin treating could significantly delay the onset, progression, and maturation of rat galactosemic cataract especially in the DG-0.1% group and then in the DG-100 group.

In this model, galactosemia resulted in a slow increasing of body weight, but lens weight consistently increased due to severe osmotic expansion especially in the early 7 days, so the two parameters were absolutely negative related. The ratio of lens weight to body weight was the key parameter of lens osmotic expansion in this galactosemic cataract model. At the end (Figure 3(b)), the ratio of lens to body weight of the control group was much higher (4.65 × 10^−4^) than the normal level (3.37 × 10^−4^); diosgenin treating (100, 200, and 0.1%) significantly inhibit the increase of the ratio at 3.28 × 10^−4^, 3.64 × 10^−4^, and 3.40 × 10^−4^, respectively.

### 3.6. AR Activity, Galactitol Level, and AR mRNA Level in Lenses

As shown in Figures 3(c), 3(d), and 3(e), AR activity was about 28.34 nmol/hr/100 mg protein, and galactitol was almost undetected in rats of the normal group. Galactosemia obviously activated lens AR (42.13 nmol/hr/100 mg protein), which leads to the accumulation of galactitol (69.84 nmol/g lens weight) and the markedly upregulated AR mRNA (2.38-fold). Three dosages of diosgenin partly restrained the above changes, especially DG-0.1% produced the strongest inhibition on AR activity (30.97 nmol/hr/100 mg protein), galactitol level (38.15 nmol/g lens weight), and AR mRNA expression (1.72-fold). These above results coincided with the effects of diosgenin on the grade of lens opacification.

## 4. Discussion

Hyperglycemia and the duration of diabetes are the major risk factors of diabetic cataract. To date, no efficient agents, irrespective of glycemic control, are utilized to treat diabetic cataract. Hyperglycemia stimulates AR enzyme, leading to the accumulation of hypertonic sorbitol and subsequent lens osmotic expansion [2], so any agent that exerts efficient AR inhibitory activity would be potential to prevent the occurrence of diabetic cataract.

At first, we optimized the screening method of ARIs. DL-glyceraldehyde can be reduced to glycerol by the AR enzyme with the conversation of coenzyme NADPH to NADP. The reaction mixture was minimized to 200 *μ*L ongoing on the 96-well ultraviolet plate, and we made all the combinations of the components. The screening results (200 nm to 999 nm) validated the observation peak of NADPH at 340 nm. The best ending method of “−20°C × 5mins” not only avoids the denaturation of AR by alkalization, acidification, and heating but also keeps a chance to remedy the inadequate reaction if needed. The only commercial ARI, epalrestat, was selected as the positive control to evaluate the accuracy of the new optimized ARIs screening method, the IC_50_ of epalrestat on normal rat lens AR was 1.32 × 10^−8^ mol/L, which was on the same level to other researches [15, 16]. Subsequently, we performed a strict ARI screening from 224 candidates including 180 natural extracts and 44 nonoriented synthetic compounds and found diosgenin as a potent inhibitor against AR. Diosgenin inhibited the normal rat lens AR in a good dose-dependent manner, and its IC_50_ was 4.59 × 10^−6^ mol/L, that was on the similar level as other ongoing plant-derived AR inhibitors [10–12].

Mechanism of cataracts caused by hyperglycemia or hypergalactosemia are similar, and they are together called “sugar cataract” [3, 17]. But their mechanism of actions is not exactly the same. AR has higher affinity to galactose than to glucose; the metabolite galactitol is more poorly reduced by SDH than sorbitol, and it takes shorter time to form rat galactosemic cataract model than another one [3, 4, 18]. So, lens osmotic expansion is more predominant in galactosemic cataract than that in diabetic cataract; the former model is more suitable for the investigation of ARIs than the latter [9, 10, 14, 19]. Therefore, we use galactose instead of glucose to induce the osmotic expansion of LECs and rat galactosemic cataract model in the following assessments.

LECs almost bear the whole metabolism of lens, so they are regarded as “life center” of the lens. AR, the key enzyme of polyol cascade, is also mainly distributed in the cytoplasm of LECs. LECs are a layer of cubic cells just located below the anterior capsule of the lens. This characteristic of LECs seems to be a double-edged sword; the sole type of LECs below the anterior capsule avoids the contamination of other cells, but the filmy anterior capsule and limited numbers of LECs bring great challenge for the primary culture. In order to enhance the adhesiveness of anterior capsule fragments to the flask bottom, we initially added only FBS without any DMEM to the flask, standing incubation lasted for 4 hours, then lied down the flask carefully. As to the osmotic stress, galactose (50 mM) could induce obvious osmotic expansion of LECs in 48 hours, plenty of tiny vacuoles gradually came into being in the cytoplasm especially around the nuclear, and AR mRNA was induced to approximate 2.85-fold of normal level. Our results showed that coincubation with DG (5 and 10 *μ*M) markedly weakened the formation of tiny vacuoles in LECs and significantly inhibited the upregulation of AR mRNA. Diosgenin could produce predominant inhibition on LECs' osmotic stress induced by galactose in vitro.

In previous studies, the galactosemic cataract was usually induced on adult rats by feeding 30–50% galactose chow; it took more than one month time to form this model [10, 14, 19]. We had recently established a steady and rapid-developing galactosemic cataract model [13]. Briefly, this sugar cataract model was induced on 21-day male SD rats by only drinking galactose solution (12.5%–10%) in 15 days. 12.5% galactose solution drank by young rats could initially induce the galactosemia and activate AR in the first 7 days, so most rats showed obvious diabetic symptoms of “three more and one less,” polydipsia, polyphagia, urorrhagia, and loss of body weight in the first stage. In order to improve the animal status, we decreased the galactose concentration to 10% from the 8th day. The downregulation of galactose concentration fully guaranteed that the mature sugar cataract could be successfully developed on galactosemic rats with good status in 15 days. In this study, galactosemic rats were treated with two different administration routes including oral taking and drinking. The addition of drinking route resulted from the following thoughts: in this model, the more galactose solution was drank by rats, the more severity was galactosemia, the less was body weight, and the less diosgenin was given by oral based on body weight; however, if we directly added the diosgenin to galactose solution, the diosgenin dosage corresponds to the intake of galactose and the severity of sugar cataract. The findings also approved the above hypothesis; the effect of the DG-0.1% group was the best among the three DG treating groups.

In vivo, sugar cataract developed more rapidly in galactosemic rats than in diabetic rats, we observed some tiny vesicles appeared in the model lens from the 3rd day, that indicated AR had already been activated by galactosemia and lens osmotic expansion came into being. We scored the lens opacification at the 6th, 9th, 12th, and 15th day, respectively. The results showed diosgenin decreased the incidence of cataract at the beginning and delayed the procession in the whole duration, but the dose-response relationship among the three DG treatment groups was not obvious. The hyperosmotic effect of galactitol then led to some unique phenomenon, body weight gain seriously lagged behind the rapid increase of lens weight, so the ratio of lens weight to body weight was the key marker, which represented the degree of lens osmotic expansion. Our results showed the ratio of the control group was significantly higher than the normal level, and diosgenin could obviously restrain the increase of the ratio in either dosage. Of course, AR activity, galactitol level, and AR mRNA of lens were also the indexes of the severity of lens osmotic stress; these results almost coincided with the above effects of diosgenin.

Diosgenin (DG), an efficient ARIs screened in present study, is the major raw materials of synthetic contraceptive and steroid hormones [20]. It is ubiquitous in many plants such as dioscoreaceae, liliaceae, and fabaceae [20]; some of them are the sources of Chinese traditional herb formulae used to treat metabolic syndrome. Recent studies have also been focused on its pharmacological effects against diabetes [21–23] and found several potential targets including *α*-amylase and *α*-glucosidase [21, 23], but the AR inhibitory effect of diosgenin and its effects against sugar cataract is a new area that has never been investigated before. DG was firstly proved to be a strong AR inhibitor in the present study, which produced obvious inhibition on osmotic expansion of LECs and delayed the formation of rat galactosemic cataract. Dietary intervention, particularly the traditional foods or herbs derived from natural sources, is always the mainstay in the treatment of diabetes. So, daily diet rich of diosgenin such as *Dioscorea nipponica* Makino, *Dioscorea zingiberensis*, *Dioscorea bulbifera*, *Dioscorea oppositifolia*, and common yam rhizome is recommended to diabetic subjects to postpone the onset of diabetic cataract, because a delay of cataract onset by 10 years can reduce the need for cataract surgery by as much as half [24]. Due to the universality of polyol pathway in pathological mechanism of diabetic complications, we also have interest on further exploring whether diosgenin and its analogs can attenuate the progression of other diabetic complications.

In conclusion, the present study demonstrates that diosgenin is a novel AR inhibitor (IC_50_ = 4.59 × 10^−6^ mol/L). In vitro, diosgenin could significantly inhibit the osmotic expansion of LECs induced by 50 mM galactose; in vivo, diosgenin could markedly delay the onset of lens opacification and maturation of rat galactosemic cataract. Therefore, we suggest that plants rich in diosgenin can be regarded as part of dietary management for diabetic patients to postpone the occurrence of sugar cataract, and diosgenin should be further investigated as a potential AR inhibitor to treat life-threatening diabetic complications.
